# Do people only adjust ongoing movements vigorously when it is advantageous to do so?

**DOI:** 10.1007/s00221-025-07082-z

**Published:** 2025-04-16

**Authors:** Eli Brenner, Melissa L. Vlasblom, Ivo Rap, Jeroen B. J. Smeets

**Affiliations:** Department of Human Movement Science, Vrije Universiteit Amsterdam, Amsterdam, The Netherlands

**Keywords:** Motor control, Online adjustments, Interception, Noise, Learning, Optimal feedback control

## Abstract

We previously found that arm movements towards a jittering target are constantly guided by the latest target position: the responses to target displacements became more vigorous as the movement proceeded, as required for the movement to reach the latest position smoothly within the remaining time. Here we examine whether this behaviour was a consequence of how that experiment was designed. We compared the vigour of adjustments in blocks of trials in which targets followed a random walk, as in our previous studies, with the vigour of adjustments in blocks of trials in which the target position varied at random with respect to a fixed position. For the random walk, the latest position is the best estimate of the final position, so neglecting earlier information can be useful. For random variability around a fixed position, the target’s position at any instant is equally informative about the final position, so making vigorous adjustments in response to the latest information is pointless. In that case, the best estimate of the final position is the average of all the encountered positions. Some participants responded less vigorously in the latter case, but most did not. We discuss why tuning the adjustments to be complete within the remaining time may be a good strategy, even when the target does not follow a random walk.

## Introduction

It is known that goal-directed arm movements can be adjusted to information that becomes available during the movement (Goodale et al. 1986; Paulignan et al. 1991; Prablanc and Martin 1992). Continuously adjusting the ongoing movement is probably fundamental to achieving the high precision that people display in goal-directed movements (Brenner and Smeets 2015a; Diedrichsen et al. 2010; Scott 2004; Todorov 2004). To study such continuous adjustments, we previously had the target of a goal-directed movement undergo multiple successive displacements as the hand moved towards it (Brenner et al. 2023). We found that the ongoing movement was continuously adjusted such that it would reach the instantaneous target position within the remaining movement time. The successive displacements in that study were completely independent of each other, so the target followed a random walk. Consequently, the latest position was always the best estimate of where the target would be in the future. Adjusting the movement with a vigour that matches what one would need to smoothly reach the latest position is therefore a sensible strategy. We found that the responses to changes in target position did indeed have something similar to minimal jerk velocity profiles (Brenner et al. 2023; Flash and Henis 1991). When the latest position is not the best estimate of where the target will be in the future, for instance because the target jitters at random around a stable position, a more sensible strategy would be to average the noisy information. This strategy would lead to less vigorous responses. Here we ask whether adjustments are indeed less vigorous when the latest position is not the best estimate of where the target will be in the future.

Another advantage of responding less vigorously to early information is that adjusting movements vigorously costs energy and can increase variability, so it can be advantageous to avoid doing so (Liu and Todorov 2007; Todorov and Jordan 2002). Indeed, people are known to adjust ongoing movements less vigorously if doing so does not interfere with their ability to successfully reach the target (De Comite et al. 2021; Knill et al. 2011; Nashed et al. 2012). For instance, arm movements are adjusted less vigorously to the hand being pushed off its trajectory when the target is wide than when it is narrow (Nashed et al. 2012). People might learn the appropriate vigour through trial and error. By reducing the vigour as long as performance is fine, and increasing it whenever they miss the target as a result of not having adjusted the movement sufficiently. That would be consistent with the difference in vigour being larger when narrow and wide targets are presented in separate blocks than when they are randomly interleaved (Orban de Xivry 2013). As there is also a difference in vigour when presented with randomly interleaved narrow and wide targets, participants must somehow be considering the instantaneous width. They might learn the appropriate vigour in a context-dependent manner (Heald et al. 2023), with target width being the relevant context. Alternatively, since people constantly reconsider the endpoint of their ongoing movements on the basis of the instantaneous circumstances (Hadjipanayi et al. 2023; Voudouris et al. 2013), the influence of the instantaneous target width might arise because the endpoint that is considered to be most suitable can change more when faced with a perturbation if the target is wider.

What would happen if reducing the vigour of responses to perturbations were not only an option, as with wide targets, but was actually advantageous? This has been examined with a manual interception task in which a moving target that participants were trying to intercept briefly deviated from its path (Brenner et al. 2022). The response to the target jumping laterally as soon as the fingertip started to move was compared across two kinds of blocks of 20 trials: blocks in which the target jumped back to its original path after 150 ms, well before the fingertip reached the target; and blocks in which it did not jump back. Participants did not respond less vigorously to the initial target jump when the target repeatedly jumped back. But it might be difficult to learn to respond less vigorously to the initial target jump, because reducing the vigour with which the movement is adjusted will probably influence the correction when the target jumps back as well, so the overall error might not be reduced. It is therefore still not clear how and to what extent the vigour with which movements are adjusted is regulated.

In the current study, the response to new information is determined by comparing arm movements after leftward and rightward target steps. We use the average difference between such movements between 100 and 200 ms after the steps as our measure of the response vigour. To examine whether adjustments are less vigorous when the latest position is not the best estimate of where the target will be in the future, we compared the vigour of adjustments in two blocks of trials. In the *random walk* block, targets followed a random walk, as in our previous experiments in which we found vigorous responses (Brenner et al. 2023; Brenner and Smeets 2023). In the *stable jitter* block, the target position jittered at random around a stable position (baseline). For the random walk block, the latest position is the best estimate of the final position, so responding vigorously to each new position estimate can be useful. For the stable jitter block, the target’s position at any instant is equally informative about the final position, so responding vigorously to the latest information is pointless. In that case, the best estimate of the final position is the average of all the encountered positions. Thus, not only is there no benefit in responding to every target displacement, but accumulating position information will even slightly improve the estimate of the final position. If one accumulates information, the response to a single perturbation will be less vigorous. If the vigour of the response depends on the benefit of responding vigorously, we should therefore find less vigorous responses in the stable jitter block.

## Methods

Fourteen adults (including three of the authors; median age 21 years old; range 20–67 years; both male and female) took part in the study. Details of the setup and calibration can be found in Brenner et al. (2023). In short, participants stood in front of a large screen (125 × 100 cm; 800 × 600 pixels; 120 Hz) onto which the starting position and target were back-projected. The room lights were on and the background was grey. The starting point was a 2 cm diameter green disk, 20 cm below the screen centre. The target was a 2 cm diameter black disk, 20 cm above the screen centre. The target was jittering laterally.

**Fig. 1:**
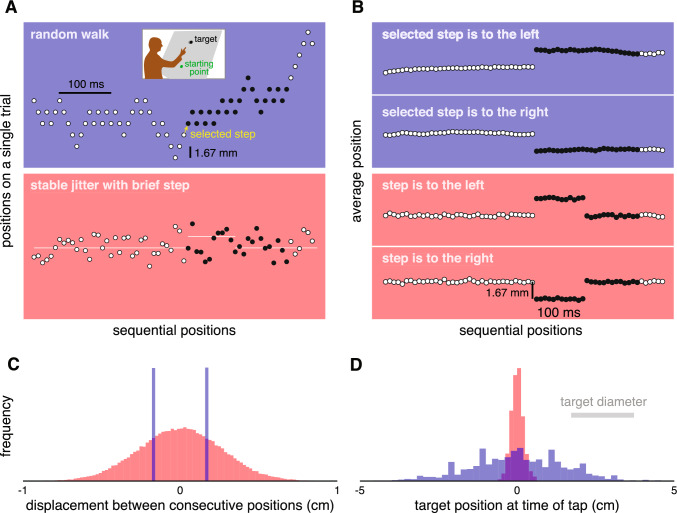
Variations in the lateral position of the target in the random walk (blue) and stable jitter (red) blocks. **A** Example trials. Dots show the target’s positions at different times during a single trial for each block. Target positions during the time that was used to determine the response are indicated by the dots being black rather than white. In random walk blocks, the target stepped 1.67 mm on each image frame, randomly either to the left or to the right. In stable jitter blocks, each new position was picked from a normal distribution around some value. This baseline value (white line) was the horizontal midline of the screen most of the time, but was briefly shifted by 1.67 mm to the left (as shown) or to the right on some trials. **B** Average target positions in each block after the trials were split into two sets (first 66 frames; 550 ms). The random walk trials were split by whether the target stepped to the left or to the right at the selected moment (yellow arrow in **A**). The stable jitter trials were split by whether the baseline value briefly stepped to the left or to the right. **C** Histogram of the displacements between consecutive target positions in the two blocks. **D** Histogram of the target positions at the moment participants tapped the screen in the two blocks. For clarity, the frequency scale differs between the two blocks in **C** and **D**

On each trial, participants had to make an upward movement from a starting point to tap on a target that jittered laterally at 120 Hz (new position every 8.33 ms) on a tilted screen (see inset of Fig. 1A). Participants were instructed to make a single movement, but they could take as long as they liked to reach the target. In the random walk block, the target followed a random walk with a 1.67 mm step size. In the stable jitter block, the target’s lateral position was selected independently for each frame from a normal distribution (Fig. 1A). Its standard deviation was 1.88 mm, so that the median size of the steps would be similar to the step size of the random walk. The mean of this normal distribution was at the centre of the screen except when it briefly shifted to quantify the response vigour (see below).

For targets that follow a random walk, we can determine the response to a 1.67 mm step at any moment by splitting the trials into two sets: one in which the step at that moment is to the left and one in which it is to the right (Brenner et al. 2023). Since the directions of the steps at all other moments are completely independent of the direction of the selected step, and are therefore as likely to be to the left as to the right, the average position will remain more or less the same after the selected step, so the positions will differ by twice the magnitude of the step between the two sets from that moment onwards (Fig. 1B). Thus, any systematic difference in the movement of the fingertip between the two sets of trials after that time can be considered to be a response to the position having changed at the selected moment. Until the selected moment, the average target positions are obviously about the same for both sets.

For targets that jitter at random around a stable baseline, we can’t directly use the response to the jitter to determine the vigour of a response to a 1.67 mm step, because the steps have many magnitudes (Fig. 1C) and consecutive steps are not independent (because the positions are sampled independently). Thus, although you could determine a response by separating trials by the direction in which the target stepped at a certain moment here too, one would only expect a very brief response because there will be no systematic difference between the sets for the next position. Moreover, it is not evident how one would equate the perturbation magnitude to that of the selected step in the random walk condition. To obtain a comparable measure of response vigour for such targets, we introduced a 1.67 mm shift of the baseline, 300 ms after the target appeared. To prevent this from introducing a benefit of relying on the latest value, the baseline returned to its original position 100 ms later. Thus, the average step size at the moment of interest is the same as the step of the random walk (1.67 mm), but the variability in the endpoints was much larger for the random walk (Fig. 1D). Most importantly, the latest position was the best predictor of future positions for the random walk, while it was no better a predictor than any other position in the stable jitter blocks. In fact, it was even a worse predictor during the 100 ms intervals in which the baseline shifted. Thus, if the vigour of the response to the steps that we introduced depends on the benefit of responding vigorously, for instance because it is learnt from previous trials, we expect to see less vigorous responses to the brief step in the baseline in stable jitter blocks than to the comparable steps in the random walk blocks. If movements are always guided by the latest information, the responses to the two kinds of steps should be similar.

The position of an infrared marker on the index finger of the participant’s preferred hand was measured at 500 Hz with an Optotrak 3020 (NDI, Waterloo, Ontario, Canada). A simple calibration procedure, involving placing the tip of the index finger on four dots on the screen, allowed us to convert measured marker positions in the Optotrak’s reference frame into positions of the fingertip relative to items on the screen. Deactivating a second measured marker whenever a flash occurred at the top left corner of the screen, and presenting such flashes whenever a new target appeared, allowed us to synchronise the measured fingertip positions with positions of the target on the screen to within 2 ms.

Each block started with 10 practice trials. After that, there were 200 trials per block. In the random walk block, all trials were the same except the random directions of the steps. In the stable jitter block, the baseline shifted to the right in 50 trials and to the left in 50 trials. In the practice trials and the remaining 100 trials the baseline did not shift. The 200 trials after the practice trials were randomly interleaved. As the practice trials and the trials in which the baseline did not shift were not used in the analysis, we had 300 trials per participant, and thus a total of 4200 trials that could contribute to the analysis. The order in which the two blocks were presented was counterbalanced across participants.

Participants started each trial by moving their finger to the starting point and keeping it there until a target appeared. The target appeared at a random moment between 600 and 1200 ms after the finger was placed at the starting point. If they moved away from the starting point before the target appeared, the target did not appear and they had to move back to restart the waiting period. Once the target appeared, they were to lift their finger off the screen and tap on the target. A tap was detected if the fingertip was within 5 mm of the screen and had an acceleration of at least 50 m/s^2^ away from the screen. If the fingertip was within the target area at the time of the tap, a sound was presented to indicate that the target had been hit, and the target was presented without jitter at its position at the time of the tap. If the fingertip was not within the target area at the time of the tap, the target moved away from the finger with respect to where it had been at the time of the tap (at 1 m/s). Thus, if the fingertip was to the right of and slightly below the target, the target moved away to the left and slightly upwards. This provided participants with accurate feedback about their errors.

**Fig. 2:**
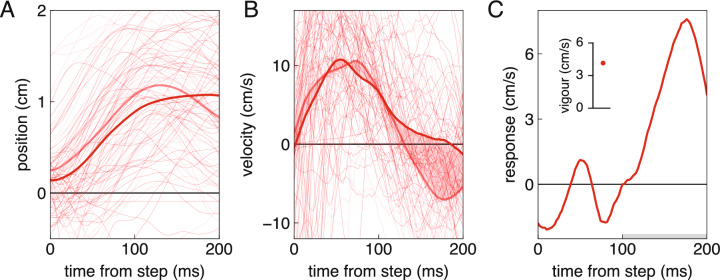
Determining the response and the vigour of the response. One participant’s data for the block with stable jitter. **A** The lateral position of the fingertip as a function of the time from when the baseline stepped to the left or to the right. Thin lines: individual trials. Thick lines: averages. The fainter curves are for leftward steps. Positive values are to the right. **B** The fingertip’s lateral velocity on the same trials. The difference between the average velocity after steps to the right and to the left (shaded area) is the response. **C** The response. Positive values are in the direction of the step. The average response between 100 and 200 ms after the step (grey bar) is the response vigour (point in inset)

To get an overall impression of performance, we determined how many targets participants hit in each block and the median amount of time it took them to do so. But we were mainly interested in how vigorously participants responded to shifts in target position. For this, we relied on determining differences between the lateral velocity of the fingertip after leftward and rightward steps. The lateral velocity was determined using a second order polynomial fit (Savitzky-Golay filter) with a window of 20 ms. Relying on differences between the movements after different steps isolates the response to the step from any other systematic lateral velocity of the fingertip (Fig. 2). For the stable jitter block, we compared the lateral velocity of the fingertip on trials in which the baseline briefly shifted to the left and to the right. For the random walk block, we compared the lateral velocity of the fingertip on trials in which the target happened to step to the left and to the right at the equivalent moment. The choice of an equivalent moment requires some thought.

Since we know that the vigour of responses to small steps in target position is larger when there is less time left in which to respond (Brenner et al. 2023), we considered the step after which there is the same amount of time left to be the equivalent moment in the two blocks. In the stable jitter block, the step in the baseline was always 300 ms after the target appeared. We determined the median time between this step and the moment of the tap, and then selected the step in the random walk block that best matched this remaining time. We did so for each participant separately, so the time from the target appearing was different for each participant (but the same for all trials by each participant).

The response is the difference between the average lateral velocity of the fingertip on trials with a rightward and leftward step (Fig. 2C). A positive response is movement in the direction of the step. Since it to takes about 100 ms to respond to a change in target position (Brenner and Smeets 1997; Brenner et al. 2023), and the step in the baseline of the stable jitter block lasted for 100 ms, we consider the responses in the two blocks to be equivalent until 200 ms after the relevant step. We therefore plot the response from the moment of the step until 200 ms after the step (black dots in Fig. 1B). We also determined the average value between 100 and 200 ms after the step for each block and participant. We use the magnitude of this average response as a measure of the *vigour* of the response (inset of Fig. 2C). This measure is used to evaluate whether the response is more vigorous for the random walk than for the stable jitter (one-sided paired t-test using the Python library SciPy).

To better understand the data, we also used the above-mentioned measure of response vigour to calculate various Spearman rank correlations (using the Python library SciPy). The first evaluated whether the response vigour in the two blocks is positively correlated across participants. The second examined whether the relationship between remaining time and response vigour that was previously found for steps at different moments is also present across participants. For each block, we checked whether the vigour is negatively correlated with the remaining time after the relevant step. We used rank correlations because we expect a monotonic, but nonlinear relationship between vigour and remaining time (Zhang et al. 2018). We used α = 0.05 for statistical significance.

## Results

**Fig. 3:**
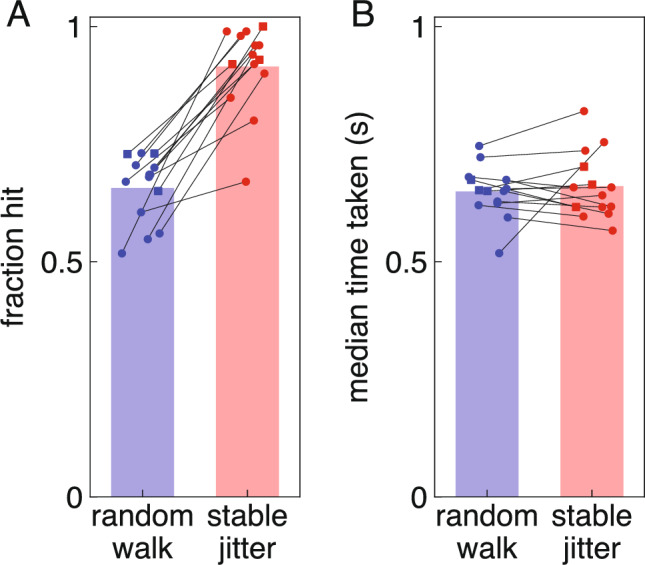
Overall performance in both blocks. Each participant is represented by two symbols, joined by thin lines. The blue and red bars indicate the mean values across participants. The three participants who responded less vigorously in the stable jitter block are indicated by squares rather than circles. **A** Participants hit fewer targets in the random walk block. **B** They did not systematically take more time to tap the screen in one of the blocks

Although the median distance between consecutive positions is similar in both blocks, within the 100 ms that it takes to respond to visual information the target will on average have moved slightly more than double the distance with a random walk than with stable jitter. It is therefore not surprising that all 14 participants hit more targets in the stable jitter block than in the random walk block (Fig. 3A). The median time they took to tap on the screen (the time from when the target appeared until a tap was detected) did not differ systematically between the two blocks (Fig. 3B), although individual participants did take different amounts of time in the two blocks.

We lost 23 of the 4200 trials due to technical failures or the participant failing to tap before the target left the screen. For determining the response, we could not use another 87 trials because the participant rotated his or her finger so much that the marker on the fingernail was not visible at some moment during the critical time (within 200 ms from the relevant step; black dots in Fig. 1), or because the participant tapped so soon that we did not have measurements during the critical time (tapping within less than 500 ms from when the target appeared in the stable jitter block, or so much faster in the random walk block than in the stable jitter block that the relevant step would have been before the target appeared). Apart from these trials (2.6% of the total) all trials were included in the analysis of the response, irrespective of whether the target was hit.

**Fig. 4:**
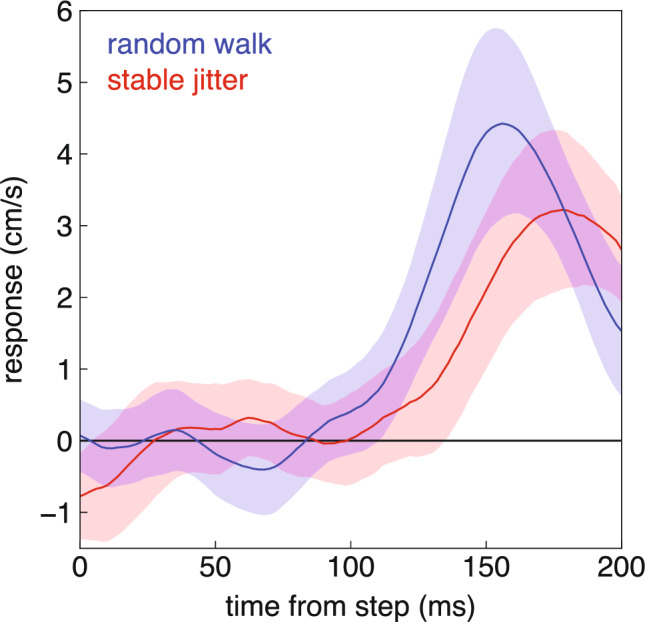
Time-course of responses to a 1.67 mm target step (with 95% confidence intervals based on the variability across participants). For the random walk, the 1.67 mm step was present on each trial (either to the left or to the right at the selected moment). For the stable jitter, the 1.67 mm is the step in the baseline; the step in individual trials varied around this value (see Fig. 1). The response is the difference between the lateral velocity of the hand after leftward and rightward steps (see Fig. 2). A positive response means that the fingertip moved in the direction of the step

**Fig. 5:**
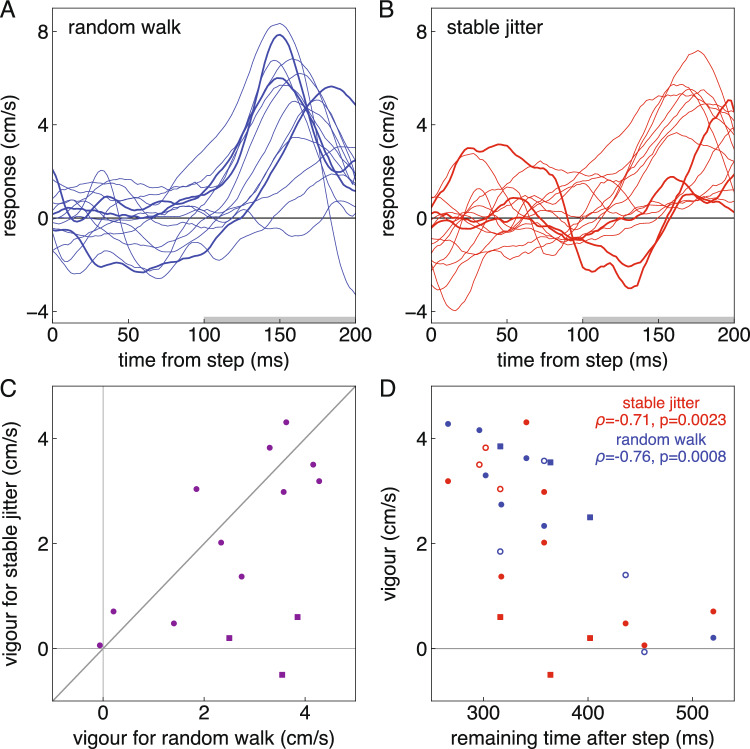
Individual participants’ responses. Each line or point represents one participant. Three participants who clearly responded less vigorously in the stable jitter block are represented by thicker lines in A and B, and by squares rather than circles in C and D. **A** Time course of the responses to the selected target step of the random walk. **B** Time course of the responses to the shift in the baseline of the stable jitter. **C** The relation between the response vigour in the two blocks. The grey bars in **A** and **B** indicate the time across which the response was averaged to obtain the vigour. **D** The relation between response vigour and the remaining time. Since the remaining time was matched across blocks for each participant, each participant’s two points are aligned vertically. The participants who hit fewest targets in each kind of block are represented by open symbols for the respective block (one participant has open symbols for both blocks; see Fig. 3A)

On average, the response appears to be slightly more vigorous and maybe to have a slightly shorter latency for the selected step of the random walk than for the shift in the baseline of the stable jitter (Fig. 4). However, within each block there is considerable variability across participants (Fig. 5A, B). The average response between 100 and 200 ms after the step was more vigorous in the random walk block than in the stable jitter block (t_13_ = 2.03, p = 0.032). But most participants who responded vigorously to the selected step of the random walk also did so for the step in the baseline of the stable jitter (Fig. 5C). Only three participants clearly responded less vigorously to the shift in the baseline of the stable jitter block (squares at bottom right). Consequently, although the correlation between the vigour in the two blocks was statistically significant, it was not very impressive (ρ = 0.49, p = 0.036). The variability in response vigour across participants that underlies this correlation is largely related to how quickly the participants tried to hit the targets, because there is a clear negative correlation between response vigour and the remaining time after the relevant step (the shift in the baseline of the stable jitter or the corresponding step of the random walk) takes place (Fig. 5D). Thus, participants’ responses were less vigorous if they had more time to adjust the movement.

Four participants clearly hit fewer targets than the others in the random walk block (Fig. 3A). Three clearly hit fewer targets than the others in the stable jitter block. These participants are indicated by open symbols in Fig. 5D. In accordance with our expectations, for the random walk blocks there is a clear negative correlation between response vigour and remaining time.

## Discussion

The aim of the study was to examine whether adjustments are less vigorous when the latest position is not the best estimate of where the target will be in the future. To find out, we compared performance in two blocks. In one of the blocks the target followed a random walk, so that its latest position was always the best estimate of where it would be in the future. In the other block the target jittered with respect to a stable baseline, so the latest position was no better for predicting the target’s future position than any other position. We found that several participants responded less vigorously when the latest position was not the best estimate of where the target will be in the future (the squares at the lower right in Fig. 5C), but most participants did not. The participants who did respond less vigorously are probably responsible for the less vigorous average response in the stable jitter blocks (Fig. 4).

A first question to discuss is whether the difference between the jitter in the two types of blocks was large enough for a change in response vigour to make any difference. We can answer this question by examining how differences in vigour relate to differences in performance for the two blocks. For the random walk blocks, good performance is not clearly related to responding vigorously: two of the participants who performed poorly might have responded slightly less vigorously than anticipated considering the remaining time (the leftmost and rightmost open blue circles), but the other two clearly did not. For the stable jitter blocks, good performance is quite clearly related to not responding vigorously: the three participants who performed poorly (open red circles) all responded vigorously (in accordance with the short remaining time), while the three participants who responded less vigorously (red squares) did not perform poorly. One participant who had close-to-zero vigour for both types of blocks performed poorly in the random walk blocks but quite well in the stable jitter ones. So, although participants could have learnt that reducing the vigour in the stable jitter blocks was advantageous, only a few did so.

Most participants responded with a similar vigour in both blocks (along the diagonal in Fig. 5C). Several participants responded considerably less vigorously in the stable jitter blocks (squares in Fig. 5D). None responded considerably more vigorously in the stable jitter blocks (there are no points at the upper left in Fig. 5C), so the considerably reduced vigour of some participants’ responses in the stable jitter blocks is unlikely to be a coincidence. It is consistent with all participants normally only considering the latest position, but some participants having learnt to accumulate information (within or even across trials) when the target jitters at random around a fixed position. Since some people even respond vigorously when it was not advantageous to do so (red points at the top left of Fig. 5D), we can dismiss the possibility that people normally accumulate information about the target’s position, but that some people learn to only respond to the latest position (and therefore respond vigorously) when the target follows a random walk. Maybe with more training more participants would have learnt to respond less vigorously to stable jitter.

If participants can learn to respond less vigorously, why would some participants respond vigorously despite it not being advantageous to do so? In our stable jitter block, we made it as beneficial as we could to not respond to the latest position in space. The latest position was not particularly predictive, and when the baseline temporarily shifted it was disadvantageous to follow the shift because the baseline always shifted back before one could tap on the target. Moreover, the variability in endpoints in the stable jitter block was small enough for participants to be able to hit the target on most trials if they would aim for the average of several recent target positions or of all previously seen positions (Fig. 1D). But the position towards which the hand is guided may not be an identified position in space (or with respect to other items; Klinghammer et al. 2015). If the hand is guided towards a position relative to oneself (Crowe et al. 2021), the target’s retinal image has to be combined with the direction of gaze, head and trunk orientation, and the position of the hand with respect to the body (Bernier and Grafton 2010; Buneo et al. 2002; Lacquaniti and Caminiti 1998), all of which may constantly be changing. In that case, any past estimate of the position will quickly become obsolete due to the participant’s own eye and body movements. The position towards which the movement is guided may therefore mainly constantly be updated to account for the participant’s own eye and body movements, rather than because the target shifts in space.

To deal with the systematic relationship between remaining time and response vigour when analysing the data, we selected steps from the random walk blocks that match the remaining time until the tap. This ensures that a difference in response vigour between the blocks cannot be the result of a change in urgency, because the time available for compensating for the relevant step is matched. We nevertheless examined whether the three participants that responded less vigorously in the stable jitter blocks differed evidently from the other participants in any way. They did not take particularly long to tap the screen in either block (Fig. 3B), so they appear to have selectively changed the vigour of their responses, rather than for instance their movement time. Although we matched the remaining time, if the three participants had taken much longer to tap the screen in the stable jitter block they might not have needed as vigorous responses because the finger was closer to the target in space. But this was not the case.

The responses that we measured were similar to those measured in earlier studies, both when considering steps of a random walk (Brenner et al. 2023; Brenner and Smeets 2023), and when considering isolated steps (Brenner and Smeets 1997; Paulignan et al. 1991; Prablanc and Martin 1992) or pairs of steps (Brenner et al. 2022; Oostwoud Wijdenes et al 2011) with no added noise. The target perturbation in our stable jitter block differs from all earlier target perturbations in that the change in target position at the critical moment was not always the same. The baseline always shifted by 1.67 mm, but the shift in the baseline and the random jitter are independent of each other, so the actual target displacement when the baseline shifts varies across trials (Fig. 1A). The response in this block is therefore determined by averaging lateral arm movement velocities of many trials with many different displacement amplitudes at the critical moment. This means that our comparison relies on the assumption that the responses to target displacements are proportional to the displacement amplitudes (Veerman et al. 2008). That appears to more or less be the case for steps such as those of the random walk block of the current study (Brenner et al. 2023). A final point to discuss is that we included the data of three authors in our analysis. We reasoned that these responses are so automatic that knowing the goal of the study could not influence the results. However, we did check that the outcome did not change if we exclude the authors. The fastest and slowest participants (Fig. 3B) were not authors. One of the three participants that responded less vigorously in the stable jitter blocks was an author.

We previously found that people adjust their movements with a vigour that will precisely bring the fingertip to the target in the remaining time (Brenner et al. 2023). In accordance with this, we here found that differences in response vigour between participants can largely be explained by how quickly the person will reach the target (Fig. 5D). Finding that some people respond vigorously to the latest target position even when it does not provide the best estimate of the target’s future position (stable jitter block) appears to be at odds with the evidence that the response to feedback does not only depend on the remaining time, but is also precisely tailored to other issues such as how vigorous the response has to be to reach the target (Crevecoeur et al. 2013; Liu and Todorov 2007). It also appears to be at odds with movements being optimized to save energy (Alexander 1997), or reduce the torque change (Uno et al. 1989) or variability (Harris and Wolpert 1998; Liu and Todorov 2007) associated with vigorous responses.

The current study differs from such studies that suggest that the vigour of responses is tuned to many aspects of the circumstances in several ways. An important difference is that people should not change the planned endpoint in the current study; considering the jitter, they should always aim for the target centre. In other studies, participants could select a different endpoint on the same target (Knill et al. 2011; Nashed et al. 2012, 2014; Orban de Xivry 2013) or a different target altogether (Brenner and Smeets 2015b; Hadjipanayi et al. 2023; Nashed et al. 2014). In some cases, the vigour of their responses was constrained by having to avoid obstacles (Crowe et al. 2023; De Comite et al. 2021; Nashed et al. 2012, 2014). Thus, it is not that the vigour cannot be modified, but presumably at each moment the natural vigour of the adjustment is set to precisely complete the required change by the end of the movement. If a new endpoint is more suitable, or obstacles need to be avoided, the trajectory is adjusted accordingly. We speculate that the participants who responded less vigorously in the stable jitter block figured out that they should move to a different endpoint (at the midline of the screen) rather than to reduce the vigour of their adjustments. This could be tested by sometimes placing the stable baseline slightly to the left or right. Or by exposing participants to the stable jitter for many more trials.

The target’s behaviour in both our blocks is quite artificial, but this does not limit the validity of our study. In daily life, random errors in judging a target’s lateral position are about 0.1° (Brenner and Smeets 2000), which corresponds to about 1 mm in our set-up. This is the uncertainty with the head fixed, but our participants were free to move, so their localisation might be less precise due to uncertainty about their own posture and movements. Most participants may therefore not have learnt to respond less vigorously in the stable jitter block because the jitter that we introduced was too small in comparison with the uncertainty of their egocentric localisation for them to benefit from responding less vigorously. It may therefore be no coincidence that at least two of the three participants who did adjust their vigour performed well in the random walk block as well as the stable jitter block (Fig. 3A). Thus, although it may be suboptimal to rely on the latest position in some specially designed situations, such as in our stable jitter block, we suspect that it might be optimal in most natural circumstances. Thus people can adjust the vigour of their responses to the circumstances, but the natural situation appears to be to respond to the latest position with the vigour that will precisely adjust the movement to reach the target within the remaining time.

## Data Availability

The data can be found at https://osf.io/gux3k/?view_only=4d25dbf4c33d463cae03380092ff5738.
